# Stigma towards dependent drinking and its role on caregiving burden: A qualitative study from Goa, India

**DOI:** 10.1111/dar.13438

**Published:** 2022-02-06

**Authors:** Sonali Kumar, Jaclyn Schess, Richard Velleman, Abhijit Nadkarni

**Affiliations:** ^1^ Addictions Research Group Sangath Community NGO Porvorim India; ^2^ Ross School of Business University of Michigan Ann Arbor USA; ^3^ Department of Psychology University of Bath Bath UK; ^4^ Centre for Global Mental Health, Department of Population Health London School of Hygiene and Tropical Medicine London UK

**Keywords:** alcohol use disorder, discrimination, India, caregiver burden, stigma

## Abstract

**Introduction:**

Stigma towards alcohol use disorders is prevalent in India and can lead to social exclusion and hamper treatment access and outcomes. Family members of individuals with dependent drinking are often their primary caregivers and play a key role in decisions around help‐seeking, treatment and recovery. The nature and role of stigma in caregiving, and the consequent burden on family caregivers of those with dependent drinking, has not been qualitatively studied in India.

**Methods:**

We conducted in‐depth interviews with: (i) men with probable alcohol dependence (*n* = 11); (ii) family caregivers (*n* = 12); and (iii) doctors with experience of treating alcohol dependence (*n* = 13) in community settings in Goa. Data were analysed using inductive thematic analysis.

**Results:**

Two primary themes were identified from the data: (i) stigma in the form of ignorance, prejudice and discrimination; and (ii) the impact of this stigma on caregiving decisions and the mental health of caregivers.

**Discussion and Conclusions:**

We found that stigma functioned as a barrier to a proper course of treatment and care, as well as a detrimental factor for caregiver's mental health and caregiving decision‐making. Stigma towards dependent drinking in the forms of ignorance, prejudice and discrimination is prevalent within homes, workplaces and health systems and might exacerbate the caregiving burden among female family caregivers. Policies, educational programs and campaigns aimed at preventing stigma in these forms would likely enable access to more inclusive and appropriate health services, benefit the health of family caregivers and improve the treatment outcomes of drinkers.

## Introduction

Alcohol use disorders (AUD) are a significant risk factor for a range of adverse health outcomes, reducing the lifespan of those who drink by more than a decade [1]. Dependent drinking is the most severe form of AUD, often characterised by increased tolerance to alcohol, impaired control over drinking, persistent drinking despite harmful consequences and physical withdrawal upon discontinuation [2]. In India, the prevalence of dependent drinking among males aged 15 and over is estimated at 7%, a significantly higher prevalence than the average for the World Health Organization South East Asia Region (2.9%) [3].

Individuals with AUDs frequently face stigma and social rejection from their communities [4]. Stigma around alcohol consumption is recognised as a key determinant of social exclusion, particularly in poorer countries [3, 5]. In India, ‘alcoholism’ is regarded with a high degree of social disapproval, probably because alcohol use is not as common among the general population (India's lifetime alcohol abstention rate is 79.2%), but is likely to be problematic among those who do drink (17–26% dependence among current drinkers) [6, 7]. A study from Goa suggests that risky drinking patterns, such as heavy episodic drinking and frequent drunkenness, are associated with lower socio‐economic class, lower educational status, perpetration of intimate partner violence, alcohol‐related fights, legal issues and workplace issues [7]. These factors probably contribute to the stigma around AUDs, which is a recognised impediment to the proper identification, diagnosis, treatment and rehabilitation of these problems [8, 9].

Stigma is not only felt by individuals with AUDs but by their families too, in the form of ‘associative stigma’ [10, 11, 12]. Alcohol problems are often perceived as a dysfunction reflective of the larger family unit, particularly in India where it is common to live with one's in‐laws in a traditional ‘joint family’ structure. Within Indian families (since alcohol dependency is more common among men), primary caregiving duties such as providing home treatment, administering medication, seeking out sources of care and monitoring treatment adherence are typically borne by the women in the home (mother, wife, sister). The caregiving role can be particularly burdensome for women, characterised by financial strain, frequent quarrels, disruptions in daily life, perceived neglect, deteriorating mental health and domestic violence [13, 14, 15, 16]. Although family caregivers are vulnerable to a range of deleterious consequences resulting from caregiving strain, they remain largely underserved by national policies and health programs. Moreover, their large role in the treatment and recovery process has not sufficiently been harnessed to improve alcohol‐related health outcomes in India.

Thus far, there is little research exploring the relationship between stigma and caregiving for alcohol use problems in India. This article reports on the nature of stigma and its role in caregiving decision‐making and burden in a community sample of men with probable alcohol dependence, female family caregivers and primary care doctors in Goa, India.

### Understanding stigma

Several approaches of understanding stigma exist. These include Goffman's [17] seminal definition of stigma as ‘an attribute that is deeply discrediting’ to Link and Phelan [18] who distinguish the processes of stigma as labelling, separating, stereotyping and status loss. Weiss *et al*. [19] recognise stigma as a ‘social process’ that includes not only the experience of devaluation or exclusion, but also the perception and anticipation of it. With regards to substance‐use related stigma, recent research has positioned stigma as both the cause and the effect of poor access to health services as it not only lowers the standard of care provided, but also lowers user willingness to access services [20]. As pointed out by Fraser *et al*. [20], stigma is not always located within interpersonal interactions but is ‘socially produced’ and often implicated within larger institutional structures and systems. Not only can this impede access to care, but it also shapes the expectations of consumers to believe that basic care and inclusion are an exception, not an entitlement [21].

For the purposes of this study, we apply Thornicroft's [22] three‐pronged framework of stigma which understands stigma as consisting of ignorance, prejudice and discrimination:

a. *Problem of Knowledge (Ignorance and Misinformation)*, or the tendency of individuals to misunderstand those with alcohol use problems, likely due to a lack of access to correct information and supportive resources. This might include common misunderstandings, for example, Individuals with AUDs are ‘weak’, ‘lazy’ or ‘not trying hard enough’ [22].

*b. Problem of Attitudes (Prejudice)* or the negative emotional responses which may accompany the thoughts that individuals may have towards those with AUDs. These emotional responses may include fear of violence, anxiety and general discomfort [22]. Stigmatising attitudes are informed by contextual factors such as negative media portrayals or insufficient government prioritisation of AUDs as a health priority [23].

*c. Problem of Avoidant or Rejecting Behaviours (Discrimination)* consisting of the *actual* behaviours which individuals may exhibit towards those with mental ill‐health or AUDs, regardless of conscious attitudes, knowledge or beliefs. Discriminatory policies and laws towards individuals with psychosocial disabilities, for example, involuntary medication or hospitalisation, reinforce discrimination at the individual level [23].

This framework was selected as it clearly locates stigma within cognitive, affective and behavioural component parts, which help to understand its underlying mechanisms and (ostensible) points of origin. Another strength of this framework is its comprehensive, research and evidence‐based approach. Although Thornicroft's [22] framework was developed for people with mental health conditions, it can be applied to substance use conditions given the repercussions of stigma for both groups at the individual, social, political and health‐care levels and the comorbidity of the two conditions. To complement the individualised focus of this framework, we recognise stigma as a function of macro‐level systems and structures which inform individual behaviour and reinforce existing social inequities (gender, class) [20, 21, 23]. Thus, we will consider the ways in which the problems identified by Thornicroft [22] might be a result of social determinants, for example, lack of available resources, limited social support, exposure to discriminatory experiences, and so on.

## Methods

### Setting

Goa is a coastal state in western India with a population of approximately 1.4 million people. Compared to many other states in India, Goa has a relatively high per capita income [24]. Alcohol is cheaper in Goa due to low excise duties and *feni*, a cashew‐based local liquor, is commonly consumed. Due to this, the prevalence of drinking in Goa is higher than in many other parts of India. Risky drinking patterns are common, with 30% of male drinkers being at least hazardous drinkers [7].

### Sample

The 36 participants comprised: (i) 11 men with probable alcohol dependence (referred to as ‘men with dependence’ hereon); (ii) 12 caregivers of males with dependent drinking (not included in the sample); and (iii) 13 general physicians from the primary health care centres who came into frequent contact with people with dependent drinking. These groups of participants were chosen to allow for a range of perspectives around dependent drinking and stigma which might later be triangulated. Men with dependence were defined as males with Alcohol Use Disorders Identification Test (AUDIT) scores of ≥20 (this positive screen for dependence was considered ‘probable alcohol dependence’, however, no diagnosis was conducted before this study) and recruited via universal screening of patients at primary health centres in Goa. The AUDIT [2] has been validated and used in India [25, 26] and a vernacular version has been used extensively in the study setting [27]. Only males with probable alcohol dependence were recruited for the study as the prevalence of AUDs among women in India is extremely low [28].

### Data collection

Structured questionnaires were used to collect demographic data (e.g. gender, age, education, marital status, employment status). Semi‐structured interview guides were then used to conduct in‐depth interviews exploring participant understandings and perceptions of AUDs, its causes and impacts, coping strategies used, help‐seeking behaviours, treatment experiences, unmet treatment needs, desired treatments and desired treatment outcomes. Interviews were not specifically focused on stigma. All in‐depth interviews were conducted in person by trained researchers.

In‐depth interviews were conducted in the vernacular (Konkani, Marathi, Hindi) or English as per participant's choice. Regular supervision was carried out with all researchers during the data collection period to check the quality of the data collected. All interviews were audio recorded and transcribed verbatim or translated and transcribed into English if in the vernacular.

### Data analysis

Two independent researchers (SK and JS) conducted a thematic analysis. The approach used was primarily inductive with no prior constructs or frameworks in mind and Thornicroft's [22] framework of stigma was applied to organise the results, in the final stage of analysis.

SK and JS first independently read through the transcripts to bring about a broad understanding of the potential themes and develop a codebook based on initial observations and notes. The reviewers then piloted the codebook using five interview transcripts, discussing discrepancies and applying revisions where needed. Transcripts were then double‐coded using the updated codebook in full using NVivo 11. Final codes were analysed by both researchers to identify common themes inductively, as supported by quotes from the interviews.

### Ethics

The Institutional Review Board at the host institution (Sangath) reviewed and approved the study within which this formative research was nested. Written informed consent was obtained individually from all participants.

## Results

The mean age of the men with dependence was 40 years with a range of 31–55 years. Five of the men with dependence had never been married. The mean age of the caregivers [Note: Demographic data was unavailable for one family caregiver.] in the sample was 45 years, with a range of 30–70 years. All caregivers were female family members; either wives (*n* = 9) or mothers (*n* = 3) of a man with dependence. The mean age for doctors in the sample was 34 years with a range of 24–61 years. Eight of the doctors were male. Table 1 shows the demographic details of participants.

**Table 1:** Demographic details of study participants

Variables	Men with dependent drinking (*n* = 11)	Caregivers (*n* = 12)	Doctors (*n* = 13)
Mean age, years (SD)	40 (7.2)	45 (12.2)	34 (9.8)
*Gender*			
Male	11	—	8
Female	—	12	5
Employed^a^	8	3	13
*Highest educational attainment*			
Illiterate	0	5	—
Primary/middle	4	4	—
High school/higher secondary	7	1	—
Graduate	—	2	13
*Relationship of respondent to man with dependence* ^b^			
Wife	—	9	—
Mother	—	3	—

^a^ Employment includes seasonal employment.

^b^ Marital status data unavailable for 5 doctors.

**Figure 1:**
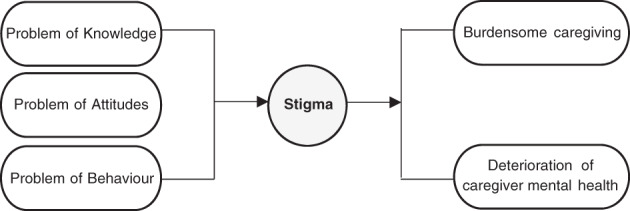
Conceptual model of the relationship between stigma, caregiving and caregiver mental health.

Two broad themes were identified from this data: (i) stigma as a problem of knowledge, attitudes and behaviours; and (ii) burdensome caregiving and the deterioration of caregivers' mental health. Figure 1 represents the relationship between these themes in a conceptual model.

### Stigma as a problem of knowledge (Ignorance and Misinformation)

Through their help‐seeking behaviours, men with dependence and caregivers demonstrated certain underlying misconceptions around dependent drinking. For example, doctors pointed out that men with dependence who appeared in their primary care centres usually sought treatment for problematic physical symptoms, rather than for alcohol use itself. This indicates a level of misinformation among caregivers and men with dependence which can result from the limited availability of educational and community‐based health programs on alcohol problems in India.

*No*, *no*, *they don*'*t come with alcohol* [dependence]*. They come with other symptoms like alcoholic gastritis. Then they get tremors sometimes… Then it*'*s abdominal pain… So they come with abdominal pain*, *gastritis*, *vomiting*. (Doctor, F, 29)

Men with dependence often relapsed after the physical symptoms were managed and brought under control. Thus, the physical symptoms in isolation, and not the dependence, seemed to be understood as the problem to be controlled.

*He was not drinking alcohol and was fine for about eight days or so and was eating well. Once he got better*, *he started drinking again*. (Caregiver, 31)

*Sometimes my drinking really gives me trouble and then I make a firm decision to stop drinking and control myself. I can do this only for about 8‐15 days. And then when I feel well and return to normal*, *I start to drink again*. (Man with dependence, 31)

Community members perceived dependent drinking to be treatable through only medical intervention, demonstrating a lack of access to appropriate information about psychosocial interventions to treat dependent drinking. Men with dependence mentioned that an ideal treatment would be a purely medical intervention, to be ‘given and eaten.’

*The treatment should cure it. They should have something to be given and eaten The treatment should include an edible item to be given at home or even from a Pharmacy*. (Man with dependence, 43)

*Tablets are sufficient enough*, *to control liquor intake*. (Man with dependence, 44)

Participants demonstrated a lack of understanding of alcohol as an addictive and harmful substance. This may have contributed to the men in this sample often unknowingly developing a drinking habit.

*Within the friend circle*, *some started to get into drinking beer. It all started with just beer. This changed after a while. A little beer is harmless they say!* (Man with dependence, 43)

Participants overemphasised the role of will power in the management of the AUD, indicating a lack of knowledge around the psychological and physiological (e.g. withdrawals) difficulties in quitting alcohol in those with dependence.

*Treatment? Only I can do it myself. When I really put my mind to it*, *I will do it. The doctors and all can*'*t do anything. I can guarantee that I will break the habit if I really put my mind to it. I can*'*t say I*'*ll listen to anyone. It*'*s not like if someone says I should stop*, *I will stop. I*'*ll stop only when I really feel like it. Even if 10 people tell me to stop this habit*, *it won*'*t help. There*'*s no need to tell me. I will stop when I feel like it*. (Man with dependence, 31)

*Yes*, *I have tried quitting for a month. At times I have quit for 15 days at a stretch. If I feel like drinking*, *then I start again. I can quit for a month or two*, *even for a year*. (Man with dependence, 45)

Men with dependence were often exposed to a triggering environment in their homes after they had quit drinking, indicating a lack of resources and knowledge within their families to better address recovery and relapse.

*Now what should I say? There are people in the family who do not quit drinking. They drink alcohol at home… His* [husband's] *father himself brings home alcohol at night and drinks… I told his father many times not to bring alcohol at home*, *but he does not listen. And at that moment he* [husband] *craves for it*. (Caregiver, 35)

Collectively, these discussions indicated that there was a ‘Problem of knowledge” within each of the interviewed groups.

### Stigma as a problem of attitudes (Prejudice)

Men with dependence and doctors reflected upon the existing prejudices around alcohol consumption. As is common with prejudice, these tags did not seem to change regardless of the individual person's behaviour, and problems of alcohol consumption were seen as a choice on the part of the drinker.

*I mean people start calling you a lazy person. They tend to call you* ‘*bevda*’ [drunkard]. *Once you get a tag of being a drunkard*, *you remain a drunkard forever*. (Man with dependence, 44)

*Alcoholic patient[s] have less of* [a] *social sense*, *that*'*s why they continue drinking. Like a normal person knows… He is doing something bad. Now every patient we tell that alcohol is bad. But they* [those with alcohol dependence] *don*'*t understand*. (Doctor, F, 27)

*That stigma is always there. You term it that* [‘alcoholic’] *and he is always an alcoholic. It's a choice so that is the problem* […] *Whatever problem he has he chose it for himself*. (Doctor, F, 24)

Caregivers perceived a loss of societal respect due to their husband's drinking and feared that their children, too, would be impacted by the prejudice around AUDs.

*I thought that people had stopped respecting me because of his drinking. I didn*'*t want my children to go through the same feeling*. (Caregiver, 35)

### Stigma as a problem of behaviour: Discrimination

Doctors and caregivers described the ways in which drinkers and their caregivers had experienced discrimination in the form of isolation within the community and a lack of care provision in treatment settings. Associative stigma around drinking was internalised as feelings of shame in caregivers and the family unit, while doctors shed light on the stigma which occurred within treatment settings.

*I feel really bad. This vice is a shame in society. We can't even sit down for a cup of tea with anyone. It's a shame for the family*. (Caregiver, 42)

*He can't be normal and decent at any moment. He's not predictable. Even if relatives or visitors come over to meet us*, *he avoids them. It looks bad. That's why no one comes*. (Caregiver, 30)

*There is so much burden of disease around us… So sometimes we* [are tempted] *to ignore alcoholism because it is lifestyle disorder. He*, *even though whatever problem he has*, *he chose it for himself. So most times doctors tend to stigmatise the person and there they might not really care. So that can cause a problem*. (Doctor, F, 24)

For men with dependence, discrimination often manifested in the form of ostracism and loss of respect within existing employment settings, or as a barrier to work opportunities.

*I have to deal with lots of people in my business. But once they come to know that you have had a drink*, *they tend to stay away. And even though they may not say anything on my face*, *I know that they speak behind my back*. (Man with dependence, 43)

*When I would drink and didn't do any work*, *people would ignore me. They didn't give me any importance and thought that I was useless. People don't care about you. They don't respect your opinions. Even if you want to make some point while speaking*, *they don't bother to listen…. Your opinions have no value*. (Man with dependence, 35)

For some caregivers, there was a lack of support and help in providing care for the men with dependence from family and community members. This ultimately enhanced the burden felt by caregivers and reflects a certain level of stigma around drinking in the community.

*We stay in a nuclear family. My mother‐in‐law stays with my brother‐in‐law. When my mother‐in‐law found out*, *she started blaming me. She pretended that she did not know anything about his* [husband] *drinking. Actually*, *they all knew about it. I then realised that there was no point in telling these people. And since I was married to him*, *I would need to take all the necessary steps in order to help him. I never involved them into these matters again. It was just amongst the two of us*. (Caregiver, 35)

*Nobody bothers… I have brought up my kids on my own. I still try to help anyone who asks me for help*, *but I don*'*t get any help from these people*. (Caregiver, 45)

Thus, the nature of stigma within this sample of drinkers, caregivers and doctors appeared to be consistent with Thornicroft's [22] framework of ignorance, prejudice and discrimination.

### Burdensome caregiving and the deterioration of caregiver mental health

The second broad theme was the influence of associative stigma on the caregiving decisions and mental health of caregivers. Associated stigma, most prominently in the form of ignorance and misinformation, may have influenced caregivers to engage in burdensome caregiving patterns.

Caregivers would sometimes try to manage the drinking problem internally until it got ‘quite late’, as one doctor described, possibly due to a lack of adequate information, little support from their families and anticipated stigma at health service points. This avoidance of help‐seeking would contribute to a worsening of the man with dependence's condition.

*What happens is we tell them*, ‘*See*, *he needs to be off alcohol*, *you need to take him*, *but they think no*, *first I will do something myself. I know he is under lots of stress*, *I know because of his job*, *or he is not having* [a] *job*, *or because of something else’. Or they will bring up somebody else*, ‘*He is having a fight with his brother*, *or he is having fight with me'*, *or* ‘*I am not properly concerned about him – that*'*s why he is doing all this’*, *or some of the other issues because of which the alcohol has started. This way they will say*, ‘*I will try my best to get him out of* [the alcohol problem]*'. So by the time they actually take them to* [district hospital] *or Alcoholics Anonymous*, *it*'*s quite late*. (*Doctor*, *F*, *24*)

Caregivers sought treatment and information first from spiritual healers and religious leaders, indicating a lack of resources on formal treatment options. This would often prove ineffective in helping their loved one quit and recover from dependent drinking.

*I went with a man from our village… This man had told me earlier that people quit drinking after taking this medicine. I was worried about the cost but then I thought I would manage it somehow. I hired a motorcycle and with the help of this man*, *went to meet this person who sold the medicine. We bowed down to him and got the medicine. But what use was it?* (Caregiver, *31*)

*Yes*, *we tried to seek a lot of divine help thinking that we had done something very wrong. We tried to seek God's blessings. I tried my level best in order to help him… For his health. But nothing worked. (*Caregiver, *45)*

In addition to informal sources of care, caregivers also sought out alternate sources such as unsolicited television advertisements, which ended up being costly and unhelpful.

*On TV*, *they show that if you pay 3000 rupees* [40 USD] *you get a cure*. (Caregiver, *42*)

*I saw it on TV at one of my workplaces and then ordered it… I ordered it on the phone number provided and they delivered it to me in four to five days. They charged me Rs.3100/‐* [42 USD] *for that. (*Caregiver, *31)*

*One of the wives told me that after watching an ad on the TV*, *she bought a medicine worth three or four thousand rupees and gave it to her husband to help him quit. She would claim that he took the medicine regularly. I met her once in the afternoon when she told me that the problem was solved. In the evening when I saw him*, *he was drunk and was walking on the road*. (Caregiver, 35)

With little support and high caregiving burden, caregivers misattributed the alcohol‐related behaviours of the man with dependence and despite all their efforts, felt neglected and undervalued.

*I tell him* [man with dependence] *to sip the alcohol from the bottle in little amounts and then gradually reduce it. But he does not listen to me. […] He does not listen to us* [family], *and he does not value us. He does not value us and therefore he does not listen to us*. (Caregiver, *31*)

*He* [man with dependence] *does not value me. I feel bad at times thinking how he does not value his own wife. I see other men who respect and treat their wives well. I do so much for him even if I am not well… He does not value me at all… That is why he values alcohol more than me*. (Caregiver, *46*)

Caregivers felt a duty to care for their loved one, unable to abandon them until they recovered. With little or no support from their community, caregivers remained isolated in their efforts, trapped in a cycle of duty and neglect. This ‘trap’ of caregiving might have contributed to the worsening health conditions of caregivers; however, they were reluctant to place their own health at a higher priority than their loved ones.

*He* [man with dependence] *does not value me at all*, *but I cannot leave him if he is not going to get better… I cannot leave him just like that. I put myself at risk and look after him. I do not understand*. (Caregiver, *46*)

*My diabetes and blood pressure got worse. But I don't care much about myself. All I care about is him. (*Caregiver, *45)*

One caregiver recognised the problematic role of associative stigma and commented on the need for educating caregivers on alcohol use disorders.

*One should explain to the wife that the there is nothing to be ashamed of. His drinking is like a sickness which ought to be treated. And that he will need all the help that he can have. So she needs to understand that. She should not feel that he is trying to degrade her by choosing alcohol over her and fight with him. (*Caregiver, *35)*

## Discussion

This article examines the nature of stigma as prevailing in a sample of men with dependent drinking, family caregivers and primary care doctors in Goa, India. We found evidence consistent with Thornicroft's [22] framework of stigma in the form of ignorance and misinformation, prejudice and discrimination. It is important to locate our findings within the macro context of the study setting, wherein there is limited education on alcohol use disorders within schools and public health campaigns, and few support structures available within the (overburdened) primary health system to address drinking. Thus, stigma (in its three forms) is a by‐product of a system with limited resources and support for patients, families and doctors to effectively prevent and address alcohol use disorders [19].

Men with probable alcohol dependence, doctors and family caregivers in this sample demonstrated a level of misinformation around dependent drinking. Stigma in this form may have contributed to short‐term, symptom‐centric treatment‐seeking and intervention‐delivery by men with dependence and doctors, delays in help‐seeking by caregivers and a triggering environment at home for men with dependence in recovery. These findings are consistent with existing literature which identifies stigma as a predictor for treatment avoidance and formal help‐seeking as a last resort by caregivers to avoid shame [14, 15]. Moreover, these findings highlight the repercussions of misinformation: it can facilitate the development of alcohol‐related problems and impede proper help‐seeking. This indicates the need for increased availability of appropriate educational and informational resources within the community, for example within national health campaigns in schools or health facilities.

Evidence of prejudice and discrimination in this sample was in the form of judgement, isolation and loss of respect from the communities of the men with dependence. They were labelled with common stereotypes attributed to drinkers, which was compounded by blame and internalisation of shame by their families. This is consistent with existing evidence that AUDs are often seen as a ‘disease of the will’, where individuals make poor choices, display a loss of control and are unmotivated for change [8, 29]. These findings underscore an environment where men with dependence and their caregivers may be subject to social and workplace ostracism and feel discouraged from formal treatment‐seeking. We found evidence of sub‐par treatment at the primary care centres in this sample including lack of staff, limited monitoring and use of physical restraints as described in Schess *et al*. [30].

Our findings were consistent with previous literature demonstrating stigma as a barrier to care [7, 31] as well as the associative stigma and isolation felt by the ‘typical’ family caregiver who is a low‐socio economic class woman [31]. Caregivers, possibly due to both self‐stigma and to a lack of resources to learn more about viable treatment options, sought out religious healers and unverified, costly medications. These help‐seeking patterns were likely exacerbated by stigmatising experiences within the formal health system, leading to anticipated stigma. Like other studies with this population, female family caregivers seemed to be ‘trapped’ in the caregiving role, unable to step out although it may be highly burdensome for them [14]. Our findings are consistent with previous literature on the nature of caregiver burden, and further highlight the way in which stigma might compound existing gender inequities tied to the woman's role as typical caregiver for alcohol use disorders [8, 19, 32].

Our findings have implications for clinical services, policy making and education in Goa. Stigma within health services indicates the need to increase mental health literacy amongst primary care doctors and nurses who are typically the first point of contact for those with AUDs. These should be carried out through mental health literacy campaigns with contact‐based strategies [33] as well as educational programs for health professionals [34]. At a policy level, our findings echo existing calls for stigma to be more adequately recognised and addressed within national policies and legislation on alcohol in Goa [35, 36]. Family caregivers must not only be included in care programs but also be targeted for treatment via education, psychosocial intervention, provision of social benefits or mental health care integrated within the public health system. Lastly, educational programs for young adults could help prevent the onset of alcohol use disorders at an early age.

This study is limited by its qualitative design, which may allow for researcher bias and limit generalisability of findings across settings. Since we did not specifically set out to explore stigma, it is possible that aspects of stigma in this community might have been missed. Thornicroft's framework is limited by its individualised lens, which overlooks the structural and social enactments of stigma [20, 21]. The socio‐economic standing of participants warranted a deeper exploration as this shapes the interaction between stigma and caregiving; see Schess *et al*. [30] for an account of the ‘poverty traps’ experienced by participants. Since our sample was limited to men, we are unable to account for the ways in which stigma impacts women with alcohol dependence and indeed the men who might care for them. Strengths of this study include its in‐depth and flexible analysis methods which allowed for themes to be organically identified from the data. The interrelatedness of the stigma (drinker centred) and associative stigma (caregiver centred) manifestations represent a significant contribution to the stigma research field and have practical implications for policy and educational programs. Although previous research does explore the impact of stigma on family caregivers, the multiple perspectives explored in this study represents a novel approach. Longitudinal, quantitative research is needed to model the causal role of stigma in caregiving patterns and burden.

## Conclusions

This article brings together two priorities in AUD care: the prevailing stigma of alcohol use disorders and the role and health of family caregivers who suffer from significant burden through the process of caregiving. By highlighting the ways in which stigma occurs within medical settings, homes, and workplaces, we are more able to develop targeted policies and community‐based programs to prevent stigma, correct misinformation around alcohol use disorders and enable increased access to care and support structures post‐care. By identifying some ways in which caregivers internalise stigma, we can develop better support structures and programs to enable caregivers to better understand alcohol use disorders and receive psychosocial support themselves.

## Conflict of Interest

The authors have no conflicts of interest.
